# Supplementary material for: Causal interplay between lactose intolerance and gut microbiota: a combined bidirectional Mendelian randomization and in vivo validation study
Source: Front Nutr. 2026 Jun 1;13:1803337. doi: 10.3389/fnut.2026.1803337 (PMC13265576; doi:10.3389/fnut.2026.1803337)
Supplement: Supplementary file 1 [file Data_Sheet_1.zip › supplementary materials/Forward/forest plot/ebi-a-GCST90027799.finngen_R12_E4_LACTONAS.pdf]

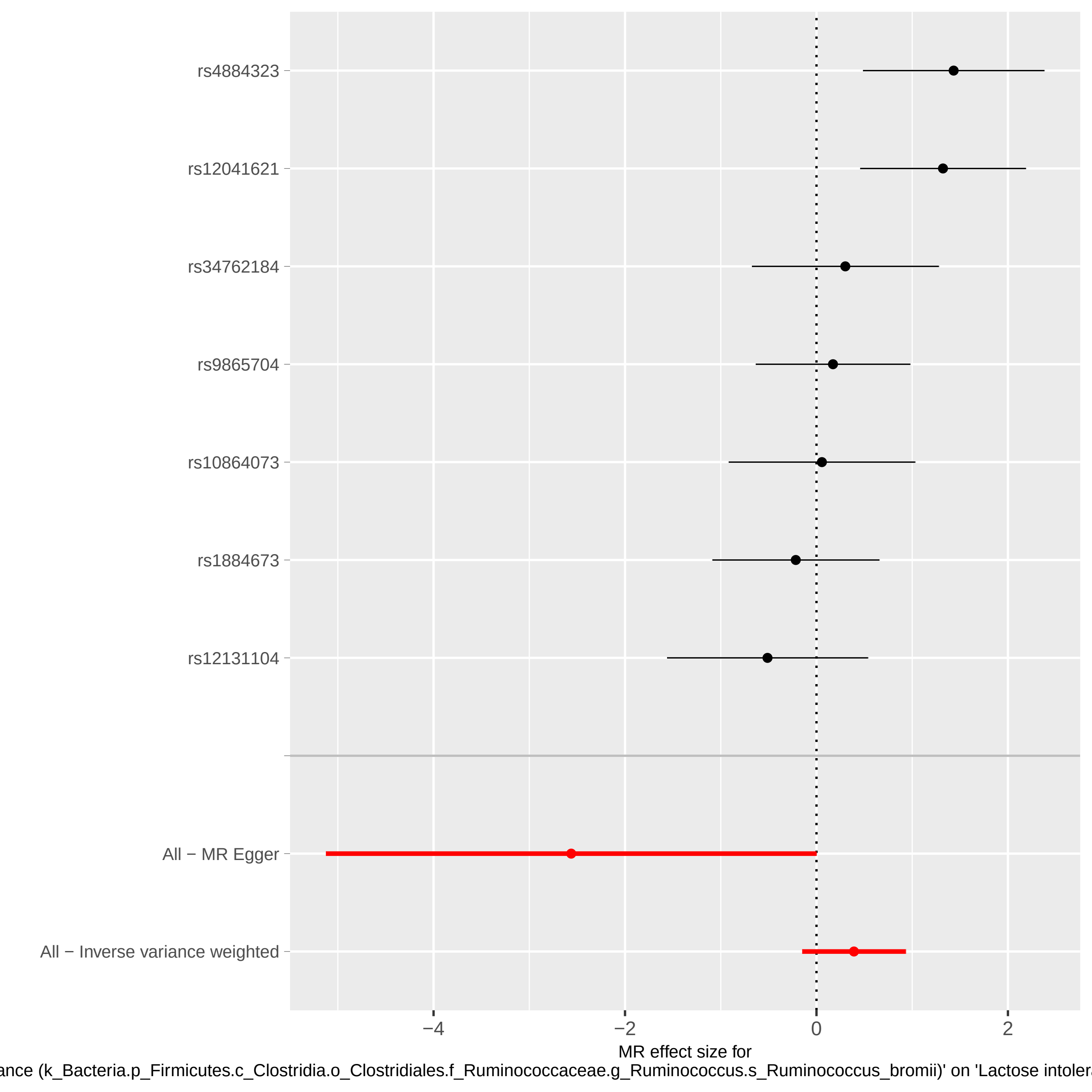

rs4884323
rs12041621
rs34762184
rs9865704
rs10864073
rs1884673
rs12131104
All − MR Egger
All − Inverse variance weighted
−4
−2
0
2
MR effect size for
ance (k_Bacteria.p_Firmicutes.c_Clostridia.o_Clostridiales.f_Ruminococcaceae.g_Ruminococcus.s_Ruminococcus_bromii)' on 'Lactose intoler
